# Inhibitors of DNA Glycosylases as Prospective Drugs

**DOI:** 10.3390/ijms21093118

**Published:** 2020-04-28

**Authors:** Grigory V. Mechetin, Anton V. Endutkin, Evgeniia A. Diatlova, Dmitry O. Zharkov

**Affiliations:** 1 SB RAS Institute of Chemical Biology and Fundamental Medicine, 8 Lavrentieva Ave., 630090 Novosibirsk, Russia; mechetin@niboch.nsc.ru (G.V.M.); aend@niboch.nsc.ru (A.V.E.); e.diatlova@g.nsu.ru (E.A.D.); 2 Novosibirsk State University, 2 Pirogova St., 630090 Novosibirsk, Russia

**Keywords:** DNA repair, DNA glycosylases, drug targets

## Abstract

DNA glycosylases are enzymes that initiate the base excision repair pathway, a major biochemical process that protects the genomes of all living organisms from intrinsically and environmentally inflicted damage. Recently, base excision repair inhibition proved to be a viable strategy for the therapy of tumors that have lost alternative repair pathways, such as BRCA-deficient cancers sensitive to poly(ADP-ribose)polymerase inhibition. However, drugs targeting DNA glycosylases are still in development and so far have not advanced to clinical trials. In this review, we cover the attempts to validate DNA glycosylases as suitable targets for inhibition in the pharmacological treatment of cancer, neurodegenerative diseases, chronic inflammation, bacterial and viral infections. We discuss the glycosylase inhibitors described so far and survey the advances in the assays for DNA glycosylase reactions that may be used to screen pharmacological libraries for new active compounds.

## 1. Introduction: Base Excision Repair

DNA in living cells is always exposed to many damaging factors of environmental and endogenous origin. These insults produce various modifications of nucleobases and lead to the formation of apurinic/apyrimidinic (AP) sites and DNA strand breaks. Accumulating damage significantly affects genomic stability and may ultimately end in mutations or cell death [1,2].

**Figure 1:**
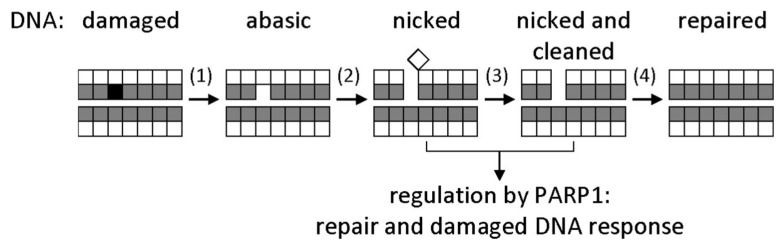
General base excision repair (BER) scheme. Bases are represented by shaded squares, and sugars by white squares. A damaged base (black) is excised by a DNA glycosylase (1); the resulting apurinic/apyrimidinic (AP) site is cut by an AP endonuclease (2); the deoxyribose fragment is removed by a deoxyribophosphate lyase (3); a correct dNMP is incorporated by a DNA polymerase, and the nick is sealed by a DNA ligase (4). Nicked DNA also activates signaling by poly(ADP-ribose)polymerase 1 (PARP1), which initiates poly(ADP-ribosyl)ation of many chromatin proteins to facilitate the access of DNA repair factors to the site of damage.

Mechanisms that correct genomic damage, commonly known as DNA repair systems, exist in all forms of life [1]. The most abundant DNA lesions—deaminated, oxidized, and alkylated bases, AP sites, single-strand breaks—are repaired by the base excision repair (BER) system [1,3]. Initially, the damaged base is recognized by a DNA glycosylase (of which, 11 are known in humans, and 8 in *Escherichia coli*), which cleaves the *N*-glycosidic bond between the nucleobase and the C1′ deoxyribose atom, forming an AP site. Afterwards, an AP endonuclease hydrolyzes the phosphodiester bond 5′ to the AP site. The repair proceeds with the incorporation of the correct dNMP by a DNA polymerase and the ligation of the remaining single-strand break (Figure 1).

DNA glycosylases belong to several major structural superfamilies and have different, sometimes overlapping, substrate specificities (Table 1). They all, however, have a common reaction mechanism: the C1′ of the damaged nucleotide is attacked by a nucleophilic moiety (either an activated water molecule in monofunctional DNA glycosylases or an enzyme’s amino group in bifunctional ones), the damaged base departs, and either an AP site is generated (monofunctional glycosylases), or a Schiff base covalent intermediate forms (bifunctional glycosylases) [4,5,6]. The Schiff base undergoes β- or β,δ-elimination and is then hydrolyzed, leaving a nick with either an α,β-unsaturated aldehyde or a phosphate as the 3′-terminal group. To access C1′, most glycosylases flip the target nucleotide from the DNA stack into the enzyme’s active site, which is equipped with a deep lesion recognition pocket, representing a convenient druggable target [7].

In human cells, BER is tightly regulated at several levels. One of the best-studied players orchestrating the BER process is poly(ADP-ribose)polymerase 1 (PARP1), together with its homologs PARP2 and PARP3, which act as nick sensors and regulate the access of repair factors to the damage sites through modification of acceptor proteins and DNA ends by poly(ADP-ribose) [8,9]. PARPs attracted attention as potential targets for cancer treatment after PARP inhibitors were discovered to be highly toxic for cells with inactivated homologous recombination repair pathway [10,11]. In human tumors, recombination repair deficiency is often associated with inactivating mutations in the *BRCA1* and *BRCA2* genes, the main driver mutations in hereditary breast and ovarian cancers. BRCA1 and BRCA2 proteins regulate the DNA break response through a pathway that does not overlap with BER [12]. Blocking both these pathways is lethal for the cell, while normal cells with active recombination repair survive PARP1 inhibition. The lethal effect of PARP inhibitors is largely mediated by PARP trapping at nicks [13,14], which mainly originate from ribonucleotides misincorporated during DNA replication [15]. Several PARP inhibitors are presently approved for clinical use, and several hundred clinical trials are ongoing.

## 2. Inhibitors of DNA Glycosylases: General Considerations

The example of PARP inhibitors highlights the concept of synthetic lethality, which underlies most of the attempts to develop BER inhibitors into practically useful drugs. Two conditions must be satisfied for such compounds to be effective. First, the target cells must experience genotoxic stress, induced either directly by DNA-damaging factors or indirectly through some kind of metabolic stress (nucleotide pool imbalance, oxidative stress, etc.). Second, if DNA damage caused by this type of stress can be repaired bypassing BER, the bypass must be blocked by mutations or another drug used in combination with a BER inhibitor. The second requirement is often satisfied in cancers, where mutations in DNA repair genes are usually among driver mutations. Genotoxic treatment may also be tuned to produce lesions repaired predominantly by BER (such as uracil accumulated through antimetabolite treatment, uracil analogs used as drugs or prodrugs, or oxidized purines appearing through MTH1 inhibition or induced by photodynamic therapy), in which case BER impairment alone could be sufficient to effect considerable cytotoxicity.

Two considerations are crucial when assessing the cytotoxic potential of DNA glycosylase inhibitors. First, unlike the enzymes underlying common BER steps, such as break signaling by PARPs or AP site cleavage by AP endonucleases, DNA glycosylases are specific for damaged bases, and their inhibition will affect only a subset of BER reactions. This in fact may be advantageous for fine-tuning or selection of concurrently used DNA damaging agents, many of which produce specific primary lesions rather than AP sites of strand breaks [16]. Second, DNA glycosylases are often ambivalent with respect to cell-killing effects of DNA damage (as discussed below in sections about specific types of lesions and their repair): they may either counteract the damage by repairing the induced lesions or potentiate the damage by converting damaged bases to AP sites or strand breaks, which are generally more cytotoxic. Thus, the inhibition of DNA glycosylases is not always warranted for inducing synthetic lethality in cancer cells or bacteria. It is always desirable to validate a particular DNA glycosylase as a drug target by knockout or knockdown approaches in an appropriate cell line or pathogen.

The inhibitors discussed in the remaining parts of this paper are mostly small-molecule compounds. Almost all DNA glycosylases are inhibited to a certain degree by non-specific single- or double-stranded DNA, competing for binding with substrate DNA [17,18,19,20,21], and a number of modified nucleotides tightly bound but not cleaved when incorporated into oligonucleotides have been described [22,23,24,25,26,27,28]. Moreover, binding and inhibition of DNA glycosylases by polyanions such as heparin [29,30,31,32] likely stems from the ability of these enzymes to bind nucleic acids. Minor-groove ligands of various chemical nature also interfere with DNA glycosylase binding [33,34]. Despite the obvious importance of such interactions for the biological functions of DNA glycosylases, delivery and targeting problems thus far prevent the therapeutic use of oligonucleotides and other macromolecular polyanions as mass action-driven inhibitors of intracellular enzymes. However, one strategy known for a while and recently applied to DNA glycosylases is the use of prodrugs that are metabolized to nucleoside triphosphate analogs and incorporated into DNA [35,36]. For example, 1′-cyano-2′-deoxyuridine triphosphate is a good substrate for DNA polymerases and, when incorporated into DNA, inhibits *E. coli* uracil-DNA glycosylase (Ung) and human UNG, displaying nanomolar *K*_i_ values [37]. Interestingly, some lesions, such as 2-deoxyribonolactone and 5-hydroxy-5-methylhydantoin [27,38,39], demonstrate an intrinsic ability to trap bifunctional DNA glycosylases covalently on DNA, reminiscent of the PARP-trapping potential of cytotoxic PARP inhibitors. Thus, the development of nucleotides that can be incorporated into DNA and trap DNA glycosylases may be an interesting direction of the glycosylase inhibitor design.

## 3. Uracil in DNA: Synergism of Glycosylase Inhibitors and Antimetabolites

Antimetabolites, the class of drugs interfering with nucleotide metabolism pathways and thereby with DNA or RNA synthesis, are one of the staples of therapeutic interventions against cancer and bacterial and protozoan infections and are especially useful in combination therapy [40,41]. Many clinically used antimetabolites, such as antifolates, interfere with thymine biosynthesis and cause the accumulation of uracil (or its analogs) in genomic DNA [42,43]. The repair of drug-induced genomic uracil is a double-edge sword: while it protects cells from the effect of this non-canonical nucleobase at low levels of substitution, extensive uracil buildup and excision are toxic and may be the primary reason of cell death after exposure to antifolates [44,45]. Therefore, the inhibition of uracil repair may have different consequences depending on the level of DNA modification and, possibly, on the nature of the modification (if different from uracil).

Human cells possess four DNA glycosylases capable of excising uracil from DNA. However, for three of them (TDG, SMUG1, and MBD4), uracil either is not the main substrate or is removed only from specific contexts (for example, methylated CpG dinucleotides). The main enzyme responsible for uracil repair, UNG, is among the most important factors limiting the efficiency of antifolates and fludarabine, whose action is based on the accumulation of uracil in genomic DNA [46,47,48]. *UNG* knockdown in human prostate cancer cell lines increases their sensitivity to H_2_O_2_ and doxorubicin [49]. Non-small cell carcinoma and lung adenocarcinoma cells develop spontaneous resistance to pemetrexed, a folic acid analog inhibiting dihydrofolate reductase, thymidylate synthase, and glycinamide ribonucleotide formyltransferase, due to a significant increase in the level of UNG, and the suppression of *UNG* expression returns the sensitivity to normal [50,51]. Some uracil analogs that are accumulated in DNA (such as 5-fluorouracil) are more toxic for cells when SMUG1, rather than UNG, is downregulated [52,53]. However, due to the structural similarity between UNG, TDG, and SMUG1, low-molecular-weight inhibitors will most likely be active against all three enzymes; therefore, the nature of the glycosylase that removes uracil during treatment with antimetabolites is not of primary importance.

UNG inhibitors in combination with genotoxic stress effectively suppress the growth of *Plasmodium falciparum* [54], *Trypanosoma brucei* [55,56], and *Trypanosoma cruzi* [57,58], which makes BER a promising target for drug intervention in protozoan infections. Importantly, some inhibitors preferentially suppress the activity of UNG from infections agents but have little effect on the human enzyme (Table 2).

Low-molecular-weight UNG inhibitors are still at a preclinical stage. The literature describes three main classes of such inhibitors. All of them are competitive, mechanism-based and mimic certain features of the transition state of the UNG-catalyzed reaction [6,79]. Free uracil and its analogs inhibit UNG enzymes from various sources, presenting submillimolar to millimolar IC_50_ [59,80,81,82,83,84,85], so successful inhibitors required extensive modification of the base to ensure tight binding. Historically, the first class of compounds active against human *Plasmodium* and herpes simplex type 1 UNGs was 6-(*p*-alkylanilino)uracils, of which 6-(*p*-n-octylanilino)uracil showed the strongest affinity, with IC_50_ ~8 μM for the viral enzyme [54,59,86,87,88] (Table 2). Bipartite inhibitors, structurally similar to the 6-substituted derivatives, consist of a uracil base or its analog linked to a phenolic or benzoic fragment [60,61,62,89] (Table 2). In the structures of bipartite inhibitors bound to UNG, the uracil part occupies the uracil-binding pocket of the enzyme, while the aromatic fragment lies in the DNA-binding groove [61,62] (Table 3). Finally, triskelion inhibitors contain three functional groups at the ends of a branched linker: either one analog of uracil and two aromatic fragments, or two analogs of uracil and one aromatic fragment [63] (Table 2). Interestingly, gentamicin, a clinically used aminoglycoside antibiotic, was reported to inhibit *E. coli* Ung [64,65] (Table 2). Although the reported IC_50_ value was quite high (0.4–1.5 mM), this effect may reflect interactions between Ung and the sugar part of DNA [21,90] and suggests another possible direction for inhibitor development.

Thymine–DNA glycosylase (TDG) has recently been validated as a possible drug target in melanoma: its knockdown causes cell cycle arrest, senescence, and cell death in melanoma cell lines but not in normal cells and prevents tumor growth in a xenograft model [91]. Screening of several mid-scale compound libraries yielded about 40 inhibitors with a variety of structures and IC_50_ >10 μM [91].

Uracil-DNA glycosylases from poxviruses (D4 according to vaccinia virus naming convention) provide a unique drug target. While they possess quite efficient uracil-removing activity, the main role of these enzymes is not in DNA repair, but in viral replication. D4 binds the A20 protein to form a processivity factor for poxviral DNA polymerases [92,93]. Deletion of the D4 gene causes a sharp drop in the ability of vaccinia virus to replicate in cells [94,95,96]. Polycyclic aromatic compounds that disrupt the D4/A20 binding interface are considered a promising class of antiviral drugs active against poxviruses [66,97,98] (Table 2).

Finally, a natural Ung inhibitor, the Ugi protein, is produced by PBS1 and PBS2 bacteriophages [99]. Although Ugi is not considered a therapeutically promising candidate, it has recently found an unexpected use in cell technologies involving CRISPR/Cas9 genome editing. A new generation of Cas9-based tools employs base editors, in which a Cas9 targeting module is fused with a cytosine deaminase to generate C→T mutations [100,101]. Repair by UNG counteracts uracil-mediated targeted mutagenesis, so co-expression of Ugi is commonly used to increase the efficiency of this gene editing procedure [100,101,102].

## 4. Oxidative Damage Repair: Key to Antibiotic Resistance?

It has been shown that BER is necessary for the survival of certain pathogenic and opportunistic bacteria (*Mycobacterium*, *Neisseria*, *Pseudomonas*, *Salmonella*) under conditions of genotoxic stress caused either by drugs or by the body’s immune response [103,104,105]. Recently, it was discovered that oxidative stress significantly contributes to the death of bacteria exposed to antibiotics of several classes. Topoisomerase inhibitors, β-lactam antibiotics, membrane-permeabilizing agents, and aminoglycosides induce the generation of hydroxyl radicals in several divergent bacterial species through an iron-dependent Fenton reaction, increasing the lethality of these drugs [106,107,108,109], whereas reducing agents such as H_2_S or NO protect bacteria from a wide range of antibiotics [110,111]. Possible reasons for the enhanced cell death include translation errors due to oxidative RNA damage [112], oxidation of the nucleotide pool followed by massive chromosome breakage at the sites of damaged nucleotide incorporation [113,114,115], and direct DNA damage by reactive antibiotic molecules, their metabolites, or reactive oxygen species [116,117]. Based on these observations, the systems of antioxidant defense and oxidative damage repair in bacteria are now regarded as promising targets for sensitization towards bactericidal antibiotics, which, if successful, can be a breakthrough in the current antibiotic resistance crisis.

In bacteria, several DNA glycosylases are responsible for oxidative damage repair. In *E. coli*, the best-studied enzymatic system, termed the “GO system” (for Guanine Oxidation), involves three enzymes: Fpg (MutM), MutY, and MutT, which have complementary functions in countering the mutagenic effect of 8-oxoguanine (oxoG) [118,119,120]. OxoG is an abundant DNA lesion that easily forms stable oxoG(*syn*):A(*anti*) Hoogsteen-type pairs, leading to characteristic G:C→T:A transversions [121,122]. Fpg is a DNA glycosylase that excises oxoG from pairs with C; such pairs appear when G is directly oxidized in DNA or when oxodGMP is incorporated opposite C during replication [123,124]. If oxoG remains in DNA and directs dAMP misincorporation, the excision of oxoG by Fpg would lead to a G→T transversion. To safeguard the cell from this mutagenic route, Fpg does not cleave oxoG:A mispairs, which are recognized by MutY, and A is excised instead of oxoG [125]. If repair DNA polymerases insert the correct dCMP opposite oxoG, the second round of repair is carried out by Fpg; otherwise, dAMP is inserted, and MutY-initiated repair is reinitiated. The third enzyme of the system, MutT, hydrolyses oxodGTP and oxoGTP to monophosphates to prevent oxoG incorporation from the oxidized nucleotide pool [126,127]. *E. coli* also possesses a homolog of Fpg, endonuclease VIII (Nei), which is not considered part of the GO system and preferentially excises oxidized pyrimidines with little opposite-base preference, although it has some activity against oxoG in vitro and prevents G:C→T:A mutations in the absence of Fpg [128,129,130,131]. Finally, endonuclease III (Nth) also removes a wide variety of oxidized pyrimidine bases [132,133].

Although the GO system has been extensively characterized in *E. coli*, little is known about its functions and the properties of its components in pathogenic bacterial species. Fpg proteins from *Salmonella enterica* [134], *Neisseria meningitidis* [135], and *Corynebacterium pseudotuberculosis* [136] have been cloned and subjected to limited biochemical characterization, which showed essentially Fpg-like properties. Several Fpg homologs from *Mycobacterium tuberculosis* and *Mycobacterium smegmatis* were characterized and found to have divergent substrate specificities resembling either *E. coli* Fpg or Nei [137,138,139,140]. For MutY, limited enzyme characterization has been done for proteins from *N. meningitidis* [141], *Helicobacter pylori* [142], and *C. pseudotuberculosis* [143,144]. The presence of a fully functional GO system with its characteristic antimutator pattern has been confirmed in vivo in their native bacterial cells for *Pseudomonas aeruginosa* [145,146], *N. meningitides* [104,135,147], *M. smegmatis* [137,148,149], and *Staphylococcus aureus* [150]. Fpg was found to be functional in vivo in *S. enterica* [134], and MutY in *H. pylori* [142].

The available information about the relevance of the GO system for the pathogenicity of bacteria shows its dual role. On one hand, it seems that this line of defense indeed assists successful primary infection. MutY deficiency has been shown to compromise mouse stomach colonization by *H. pylori* [142]. Successful macrophage infection by *Brucella abortus* requires intact *fpg* and *mutY* genes [151], and *M. tuberculosis* Fpg and Nei are required for lung colonization in a rhesus macaque model [152]. Hypervirulent *Neisseria* isolates maintain functional *fpg* and *mutY* despite having a general mutator phenotype [147]. On the other hand, hypermutability associated with GO system inactivation sometimes provides the variance for selection of highly virulent or drug-resistant strains [153,154,155,156]. This underscores the importance of a thorough characterization of the GO system for a given pathogen to assess it as a possible drug combination target.

Human homologs of Fpg and Nei (NEIL1, NEIL2, and NEIL3) are significantly different from the bacterial proteins in their sequence and structure, making realistic the development of small ligands selectively targeting the bacterial enzymes. MutY and Nth appear to be less selective targets.

Few specific inhibitors of the bacterial GO system have been reported. Fpg is weakly inhibited by free damaged base analogs such as 2,6-diamino-4-oxo-5-(*N*-methylformamido)pyrimidine, 5-nitroso-2,4,6-triaminopyridine, and 5-nitroso-4,6-diamino-2-oxopyridine [67,157]. The base analog with the strongest ability to suppress Fpg activity (IC_50_ ~10–100 μM) is 2-thioxanthine (Table 2); however, it is not a true inhibitor but rather a reagent that oxidizes cysteines in the zinc finger in Fpg, Nei, and NEIL2 [67,68]. MutY from *C. pseudotuberculosis* was reported to be sensitive to suramin, an antiprotozoan and anthelmintic drug that acts intracellularly [32] (Table 2); however, the relevant drug uptake pathways are likely absent in bacteria. Interestingly, Fpg is strongly inhibited by Cibacron Blue F3GA, a dye bearing structural resemblance to suramin [69] (Table 2). Several papers described the inactivation of Fpg, Nth, OGG1, NEIL1, and NEIL2 by NO-producing agents and suggested damage to redox-sensitive groups in the enzyme molecules [158,159,160,161,162]. However, the mechanisms of this reaction remain unclear, since the known redox-sensitive groups in these glycosylases are different or absent altogether. A screen of a natural product library revealed several inhibitors of *M. tuberculosis* Nei2, the best of which, norlobaric acid, has *K*_i_ = 74 nM [70] (Table 2).

## 5. Oxidative Damage Repair: Cancer Sensitization Strategy

OGG1 is a human DNA glycosylase that initiates the repair of oxidized purine bases, mainly oxoG and formamidopyrimidines, thus being a functional analog of Fpg. Nevertheless, in its sequence and structure, OGG1 is completely different from Fpg (Table 1). Overexpression of *OGG1* in fibroblasts, pulmonary epithelial cells, and bone marrow protects them from the toxic effects of thiotepa, carmustine, and mafosfamide, which mainly yield *N*7-alkylated purines further hydrolyzed in the cell to formamidopyrimidine derivatives [163,164,165]. However, it is unclear whether normal OGG1 levels can reduce the toxicity of these drugs in tumor cells. A similar effect of OGG1 has been described for cisplatin and oxaliplatin [166], although the nature of the damage removed in this case is not entirely clear. Of the antitumor agents that produce oxidative DNA damage, *OGG1* downregulation or inhibition sensitizes cells to bleomycin [167] and ionizing radiation [168].

As with uracil incorporation and repair, the activity of OGG1 may not only safeguard cells from genomic damage but also potentiate the action of DNA-damaging agents, converting damaged bases to more cytotoxic strand breaks. For instance, OGG1 downregulation protects several cancer cell lines from β-lapachone, an NAD(P)H dehydrogenase (quinone 1)-dependent redox cycling drug that produces copious amounts of intracellular H_2_O_2_ [169]. Hence, a strategy alternative to OGG1 inhibition may consist in saturating the BER capacity with oxidative lesions. In human cells, OGG1 together with mismatched adenine-DNA glycosylase MUTYH and nucleoside triphosphatase MTH1 (NUDT1) forms an analog of the GO system, which prevents the mutagenic effect of oxoG [170]. Recently, the knockdown or inhibition of MTH1, which hydrolyzes oxoG triphosphates and prevents their incorporation into growing DNA and RNA chains, was shown to be toxic to tumor cells, due to the accumulation of oxidized bases and DNA breaks [71,171,172]. Apparently, as in the case of PARP inhibitors, selective toxicity is due to the suppression of the last remaining pathway for the oxidative damage repair in cancer cells. Several low-molecular-weight MTH1 inhibitors were identified, including a clinically approved tyrosine kinase inhibitor, crizotinib [71,171,173] (Table 2). Interestingly, crizotinib possesses a chiral center that gives rise to *(R)*- and *(S)*- enantiomers, of which clinically used *(R)*-crizotinib inhibits c-MET and ALK protein kinases, whereas the *(S)*-enantiomer preferentially binds to and inhibits MTH1 [71,174,175]. While it is still debated to which extent the cytotoxic activity of crizotinib and other MTH1 inhibitors is dependent on MTH1 and oxidative overload [172,176,177,178], most reports agree that oxidative damage is an important cell-killing factor, although its causes might not be limited to a direct suppression of MTH1 activity (recently reviewed in [179]). As of today, (*S*)-crizotinib is not used for patient treatment. However, another anti-cancer drug candidate, karonudib, was developed from previously found MTH1 inhibitors [172,180,181]. Presently, two Phase 1 clinical trials of karonudib are registered with the US National Library of Medicine Clinical Studies Database.

Inhibitors of OGG1 have been reported in the literature but have not yet reached clinical trials. Mechanism-based approaches had only limited success: both oxoG base and its analogs proved to be weak inhibitors [182,183], while substituted 2,6-diaminopurines performed slightly better [76]. Experimental and computational screening of small-molecule pharmacological libraries produced several hits that were expanded into inhibitors with submicromolar affinity, structurally unrelated to the OGG1 substrate [72,73,74] (Table 2). Combinatorial design based on the identified OGG1 and MTH1 inhibitors was used to obtain a compound with submicromolar affinity for both these enzymes [75] (Table 2).

Other DNA glycosylases that repair oxidative damage, including NEIL1, NEIL2, NEIL3, MUTYH, and NTHL1, have been targeted less successfully. Purine-analog library and general library screening produced several inhibitors for NEIL1, but their affinities were in the micromolar range, and the target selectivity was quite low when compared with the inhibition of other glycosylases [76,184] (Table 2). Fumarylacetoacetate was reported to inhibit NEIL1 and NEIL2 and to a lesser degree, OGG1 and NTHL1 (Table 2), but the structural reasons under this effect have not been established [77]. Moreover, these enzymes have low experimental support as targets for sensitization to antitumor therapy, although NEIL1 confers some resistance to ionizing radiation and antifolates [185,186].

## 6. Oxidative Damage Repair: Unexpected Connections

While DNA damage and its repair are well understood in the cancer paradigm, two unexpected connections of oxidative damage with other human pathologies emerged recently. OxoG and its repair by OGG1 are suspected to play a regulatory role in the inflammatory response. Several lines of evidence support this conclusion. *Ogg1*-null or -depleted mice show a significantly alleviated inflammatory response to many factors, including bacterial endotoxins, *Helicobacter* infection, foreign protein response, ragweed pollen grain extract-induced allergy, etc. [187,188,189,190]. Interestingly, however, the inflammation associated with UVB or pulmonary hyperoxia is enhanced rather than reduced in *Ogg1*^−/−^ mice [191,192,193], suggesting that OGG1-dependent inflammation requires foreign antigens. After the excision of oxoG, the OGG1·oxoG complex can bind Ras family GTPases and facilitate the GDP-to-GTP exchange [194,195], which triggers the signaling pathway leading to the activation of NF-κB, the key pro-inflammatory transcription factor [196]. Moreover, OGG1 can bind oxidized G-rich promoters of pro-inflammatory genes in an enzymatically non-productive mode and facilitate their expression by attracting NF-κB [197,198,199]. A small-molecule OGG1 inhibitor, TH5487, was developed that competes with oxoG for binding and downregulates the inflammatory response in a mouse model [73] (Table 2). Although TH5487 has a 4-bromobenzimidazolone moiety, which is structurally similar to oxoG, the crystal structure of its complex with OGG1 [73] (Table 3) unexpectedly revealed that the oxoG-binding pocket is occupied by another moiety of TH5487, *p*-iodophenylacetamide, whereas 4-bromobenzimidazolone resides in a so-called *exo*-site, which normally binds undamaged G and serves as a transient binding site for oxoG on its way to the active site [200,201]. Thus, TH5487 functionally resembles bipartite inhibitors of UNG, simultaneously engaging two selective binding sites in the enzyme molecule. Such design may be employed to construct new potent inhibitors of OGG1 and other DNA glycosylases.

In addition, inhibition of OGG1 holds promise to prevent or delay the onset of Huntington’s disease in risk groups. This hereditary condition, which belongs to the class of “trinucleotide repeat” genetic diseases, is caused by expansion of the (CAG)_n_ repeat run in the *HTT* gene beyond the critical length of ~35 repeats [202]. Before becoming symptomatic, carriers of a pathogenic allele experience an explosive growth of the (CAG)_n_ run up to several hundred repeats in the striatum at the early stage of the disease [203]. This expansion is triggered by the normal repair of oxoG initiated by OGG1 [204,205] and is likely caused by an imbalance of BER enzymes in this part of the brain, which leads to the accumulation of unprocessed repair intermediates [206]. In the Huntington’s disease mouse model, *Ogg1* knockout suppresses the repeat number growth in the striatum and delays the onset of motor dysfunction [207]. Thus, in carriers of the pathogenic *HTT* allele, for whom the penetrance is inevitably 100%, inhibition of OGG1 may be a reasonable therapeutic strategy.

## 7. Alkylation Damage Repair: Dual Consequences

Alkylating antitumor agents produce many damaged bases, including *O*^6^-alkylguanine repaired by *O*^6^-methylguanine–DNA methyltransferase (MGMT), and ring-alkylated purines repaired predominantly by BER [16,208]. Unlike other DNA glycosylases, which impart resistance to DNA-damaging agents to cells, *N*-methylpurine-DNA glycosylase (MPG, alias alkyladenine-DNA glycosylase (AAG), and alkylpurine-DNA *N*-glycosylase, APNG) may increase the cytotoxicity of alkylating antitumor agents, removing alkylated bases from DNA to form AP sites, which are more dangerous for the cell [208,209,210,211,212,213,214]. A similar sensitization mechanism is also characteristic of UNG, TDG, and MBD4 DNA glycosylases when they repair C5-halogenated uracil derivatives [215,216,217,218]. On the other hand, inhibition of MPG in carcinoma cells sensitizes them to alkylating agents [219], and *Mpg*^−/−^ murine cells are hypersensitive to 1,3-bis(2-chloroethyl)-1-nitrosourea and mitomycin C (but not to alkylating nitrogen mustards) [220]. An integrative model of temozolomide-induced DNA damage and DNA repair by MGMT and MPG in glioblastoma predicts that inhibition of both enzymes is the most successful sensitization strategy [221]. For temozolomide-resistant forms of glioblastoma, the combination of inhibition of BER enzymes and PARP-dependent signaling is effective [212,222]. In addition to the detoxification of anticancer drug adducts, MPG and OGG1 have been reported to hydrolyze a human cytomegalovirus replication inhibitor, 2-bromo-5,6-dichloro-1-(β-d-ribofuranosyl)benzimidazole, opening the possibility of antiviral action of drug combinations including DNA glycosylase inhibitors [223]. Bacterial *alkA* mutants are hypersensitive to methyl methanesulfonate [224,225]; however, alkylating agents are not among clinically used antibacterial drugs, so this vulnerability is hard to exploit.

Alkylbase-removing DNA glycosylases are the least explored group in terms of specific inhibitors. N3-substituted adenine derivatives are competitive inhibitors of bacterial Tag and mammalian MPG [226,227,228,229,230]. Based on this observation, a series of structural analogs has been computationally designed to inhibit TagA from *Leptospira interrogans*, the infectious agent of leptospirosis, although no experimental evidence was provided for their activity against the enzyme or the pathogen [231]. A natural flavonol, morin, inhibits MPG [78] (Table 2).

## 8. Assays for DNA Glycosylase Activity

Most basic research on DNA glycosylases was and is still done using radioactively labeled oligonucleotides and analyzing substrates and product by gel electrophoresis. While this assay offers the highest sensitivity, it is labor-intensive, not easily scalable, and inconvenient when screening pharmacological libraries. In the past years, a number of fluorescence-based assays to follow glycosylase activities appeared, some of them coupled with signal amplification to increase the sensitivity.

**Figure 2:**
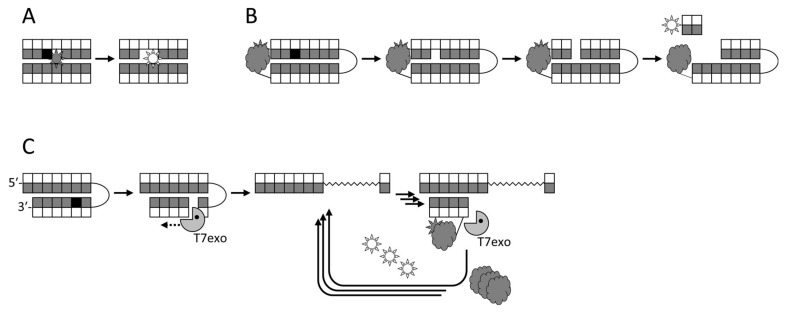
Schemes of several fluorescence-based approaches to DNA glycosylase detection. (**A**), fluorescent reporters adjacent to the lesion; (**B**), molecular beacons with a fluorophore–quencher pair; (**C**), T7 exonuclease-assisted signal amplification due to cyclic degradation of a probe bearing a fluorophore–quencher pair.

The first attempts to utilize fluorescent labels for detecting DNA glycosylase activities in a homogeneous mode were based on changes in the signal from a fluorophore incorporated next to a lesion upon the eversion or the excision of the damaged base [232,233] (Figure 2A). Although this approach has been used for inhibitor screening [75], it is not very sensitive, and fluorophores adjacent to a lesion may even inhibit the measured activity. Molecular beacon substrates developed later consist of an oligonucleotide hairpin or a duplex bearing a fluorophore and a quencher at its termini (Figure 2B). Such substrates allow measuring DNA glycosylase activities both in vitro and in living cells [76,234,235,236,237] and have been used in glycosylase inhibitor library screening [73,76]. In this case, the glycosylase must be bifunctional to nick the substrate, or else an AP endonuclease has to be present in the assay. Several types of arrays or beads with immobilized damaged oligonucleotides have been reported, in which only the damaged strand is labeled, and the cleavage produces short DNA fragments that can be washed off [238,239,240,241,242]. While such assays are not homogeneous, they are well suited for multiplexing and parallel screening. Fluorophores can also be incorporated into double-stranded DNA in situ through base excision, AP site cleavage, and gap filling by DNA polymerase β with a labeled dNTP [243].

An interesting approach was suggested that employs a DNAzyme inactivated by a strategically placed U residue, and the excision by uracil-DNA glycosylase reactivates the DNAzyme, which cleaves a fluorescent substrate [244]. However, it cannot be applied to other glycosylases that require double-stranded substrates. In a more advanced version, base excision generates a specifically folded quadruplex, which forms a fluorescent complex with quadruplex-selective ligands [245,246,247].

The most sensitive fluorescent assays rely on the formation of a nick after the cleavage by a DNA glycosylase (either bifunctional or coupled with an AP endonuclease), followed by signal amplification. The amplification may be exonuclease-assisted linear isothermal, in which a beacon is annealed and degraded by a double-stranded specific exonuclease in repeated cycles [248,249,250] (Figure 2C). Alternatively, the signal may be enhanced by rolling-circle amplification, using any suitable assay to detect the newly synthesized DNA [251,252,253,254]. Finally, nick formation can serve as a starting point for exponential isothermal amplification [255,256] or cascade hybridization [257].

## 9. Conclusions

DNA glycosylases, as enzymes that initiate base excision repair, represent an attractive pharmacological target. Their structures reveal mechanism-based features, such as deep pockets for substrate base binding, indicating potential druggability, and several successful attempts of library screening produced tantalizing leads that can be explored further to develop drugs for cancer and infectious diseases. Moreover, recent findings implicate DNA glycosylases not only in genome protection but also in regulatory pathways and suggest that they can be targeted in some inflammatory and neurodegenerative processes. A number of rapid and sensitive assays for screening DNA glycosylase activities were developed in the past few years, which should facilitate the search for their inhibitors.

The most important factor that complicates the targeting of DNA glycosylases in the now well-established framework of synthetic lethality, e.g., in cancer therapy, is their dual function in cell killing. On one hand, glycosylases initiate the repair of genotoxic adducts and, in theory, should potentiate their action. On the other hand, there are many cases in which the main lethal lesions are not adducts per se but intermediates of their repair, such as AP sites or DNA breaks. Such intermediates usually accumulate if the activity of downstream BER enzymes are insufficient to process the inflicted amount of genomic lesions in full. In these situations, DNA glycosylase inhibition would protect rather than sensitize cells to genome damage. Optimally, DNA glycosylases should be targeted in some form of precision therapy, based on the general model of toxicity of various adducts and the specific knowledge of adduct spectra and downstream BER capacity in the affected cells.

Outside of the cancer field, DNA glycosylase inhibition is most likely to find its soonest clinical application in antiviral therapy, since two important groups of human pathogens, poxviruses and herpesviruses, possess their own uracil-DNA glycosylases, a validated target required for replication in host cells, and several promising drug leads are available. Inhibition of OGG1 to prevent somatic trinucleotide repeat expansion in Huntington’s disease also has high priority due to the extreme morbidity and mortality of the condition and the lack of other drugs, although lead compounds capable of brain delivery have not been reported so far. The inflammation-modulating action of OGG1 inhibitors, albeit attracting considerable attention, would still require much research and mechanistic insights to produce drugs comparable with more traditional anti-inflammatory agents. Even if more basic research is required to validate DNA glycosylases as targets for antibacterial combination therapy, yet the payoff in this area may be the largest one. The prospects of bringing DNA glycosylases into the circle of drug targets ultimately depend on our understanding of their action in DNA repair and connection with other cellular pathways.

## Figures and Tables

**Table 1:** Examples of DNA glycosylases found in humans, *Escherichia coli*, and other species.

Structural Superfamily	*E. coli*	Human	Other Organisms	Major Substrate Specificity
α/β fold	Ung	UNG		U in any context
	Mug	TDG		U, T, 3,*N*^4^-ethenocytosine, oxidized/deaminated 5-methylcytosine opposite G
		SMUG1		U:G
helix–hairpin–helix		MBD4		U opposite G in CpG context
	Nth	NTHL1		oxidized pyrimidines
		OGG1		oxidized purines
	MutY	MUTYH		A opposite 8-oxoguanine
	AlkA			ring-alkylated purines, 1,*N*^6^-ethenoadenine, hypoxanthine
			*Micrococcus luteus* Pdg	cyclobutane thymine dimers
helix–two-turn–helix	Nei	NEIL1		oxidized pyrimidines
		NEIL2		oxidized pyrimidines in DNA bubbles and loops
		NEIL3		oxidized pyrimidines in single-strand DNA
	Fpg			oxidized purines
	Tag			3-methyladenine
			T4 phage DenV	cyclobutane thymine dimers
HEAT repeats			*Bacillus cereus* AlkC, AlkD	ring-alkylated purines, minor groove adducts

**Table 2:** Properties of selected DNA glycosylase inhibitors discussed in this review.

General Structure	R	Enzyme	Species	I_50_, μM	Reference
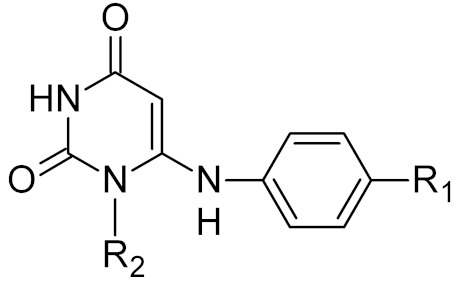	R_1_ = *n*-octyl R_2_ = H	UNG	HSV1	8	[59]
			human	>300	
	R_1_ = *n*-octyl R_2_ = 1-methoxyethyl		*Plasmodium falciparum*	17	[54]
			human	>160	
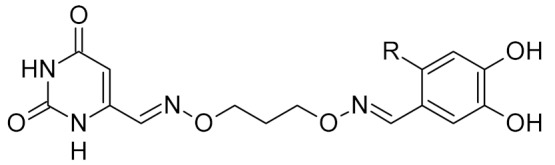 bipartite inhibitors	H	UNG	human	1.1	[60]
	OH			0.26	
	F			2.7	
	Cl			16	
	Br			40	
	NO_2_			40	
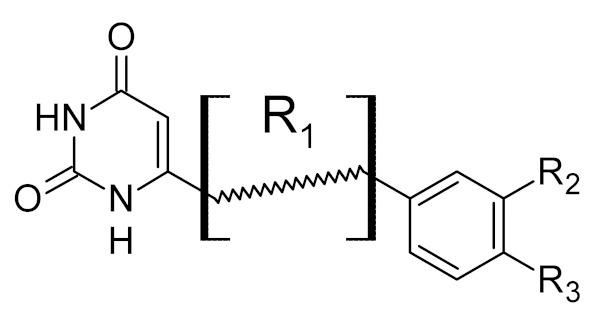 bipartite inhibitors	R_1_ = 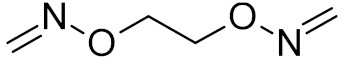	UNG	human	40	[61]
	R_2_ = COOH, R_3_ = H				
	R1 = 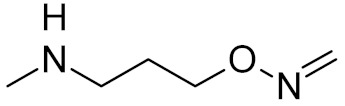			1.6	
	R_2_ = COOH, R_3_ = H				
	R_1_ = 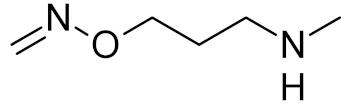			100	
	R_2_ = COOH, R_3_ = H				
	R_1_ = 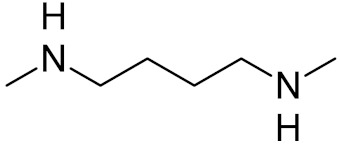			315	
	R_2_ = COOH, R_3_ = H				
	R_1_ = 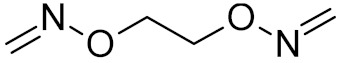			6	[62]
	R_2_ = H, R_3_ = COOH				
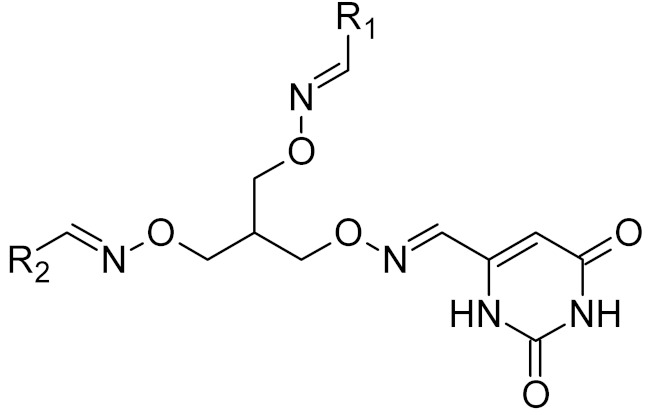 triskelion inhibitors	R_1_ = R_2_ = 3,4-dihydroxyphenyl	UNG	human	1.6	[63]
	R_1_ = 6-uracil R_2_ = 3,4-dihydroxyphenyl			0.9	
	R_1_ = R_2_ = 3-carboxyphenyl			1.7	
	R_1_ = 6-uracil R_2_ = 3-carboxyphenyl			0.9	
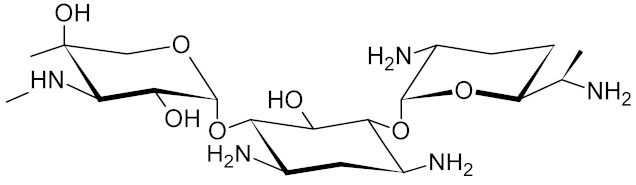 gentamicin		UNG	not specified	1500	[64]
			*E. coli*	420	[65]
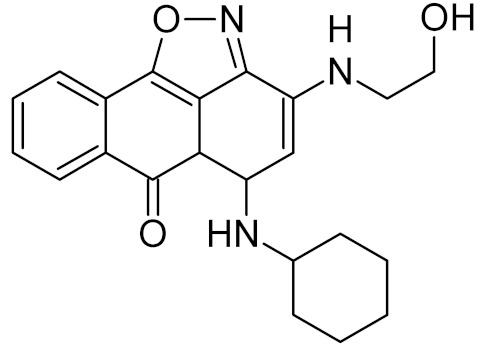		UNG	vaccinia virus	34 *	[66]
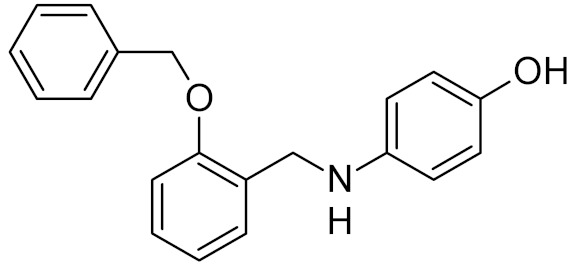				5.1 *	
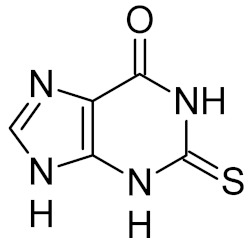 thioxanthine		Fpg	*E. coli*	17	[67]
		Fpg	*Lactococcus lactis*	100	[68]
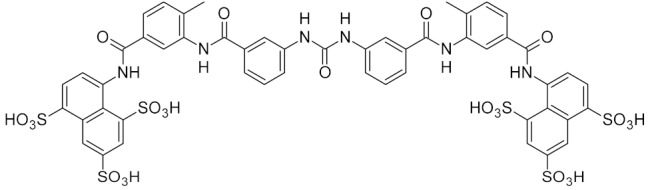 suramin		MutY	*Corynebacterium pseudotuberculosis*	16 **	[32]
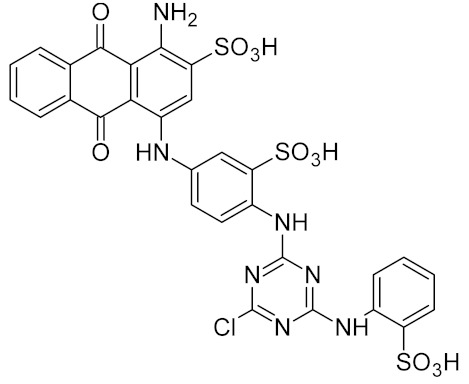 Cibacron Blue F3GA		Fpg	*E. coli*	0.005 **	[69]
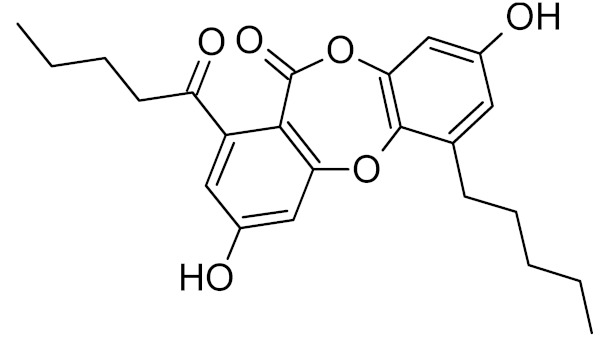 norlobaric acid		Nei2	*Mycobacterium tuberculosis*	42, 0.074 **	[70]
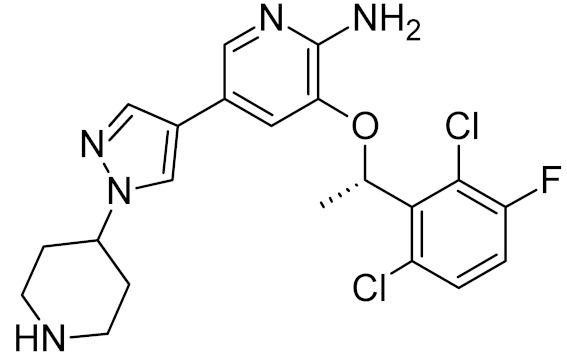 *R*-crizotinib		MTH1 ***	Human	0.33, 0.048 **	[71]
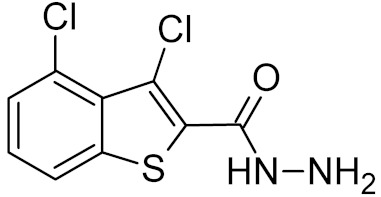		OGG1	human	0.22	[72]
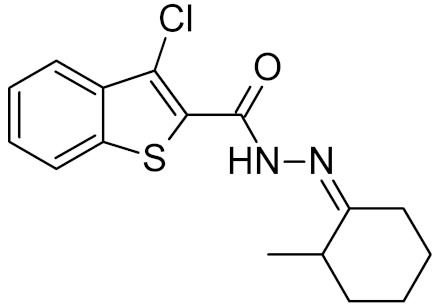				0.27	
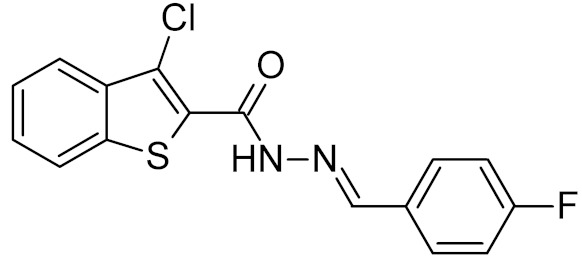				0.33	
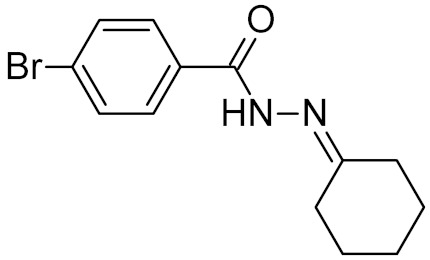				0.63	
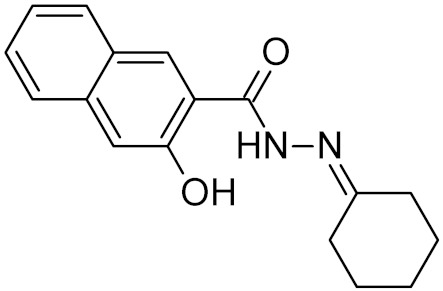				0.34	
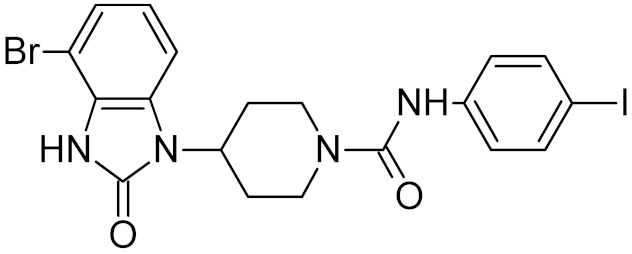 TH5487		OGG1	mouse	0.34	[73]
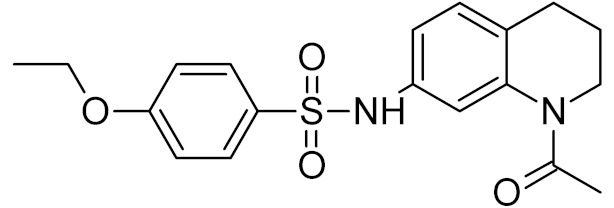		OGG1	human	2	[74]
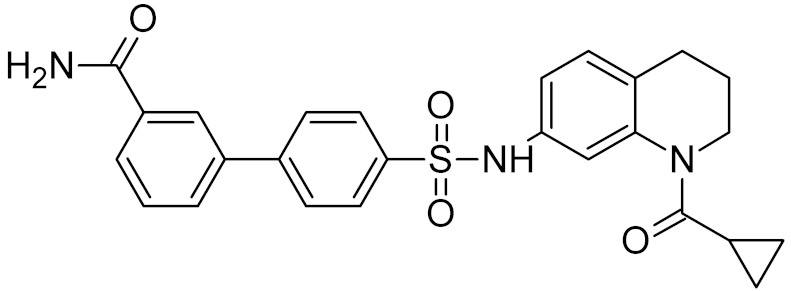				0.059	[74]
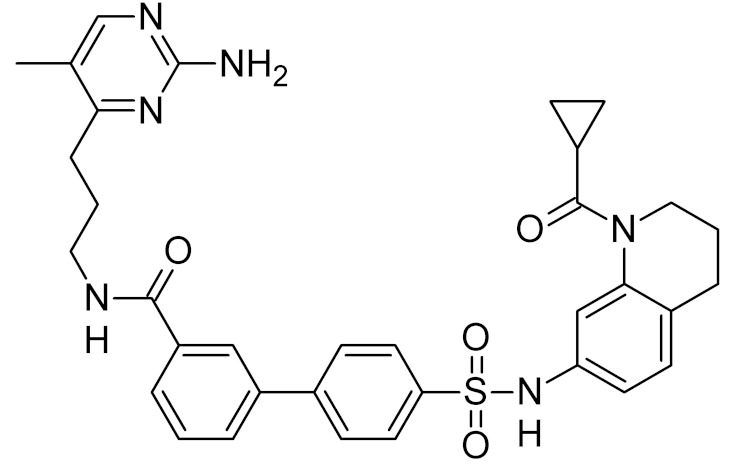 SU0383		OGG1	human	0.49	[75]
		MTH1 ***	human	0.034	
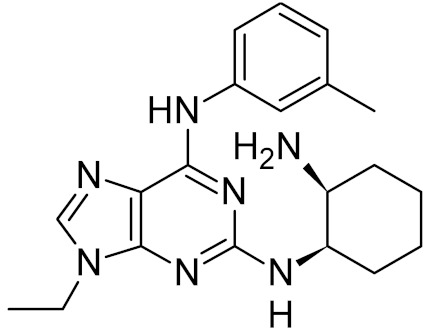		NEIL1	human	25	[76]
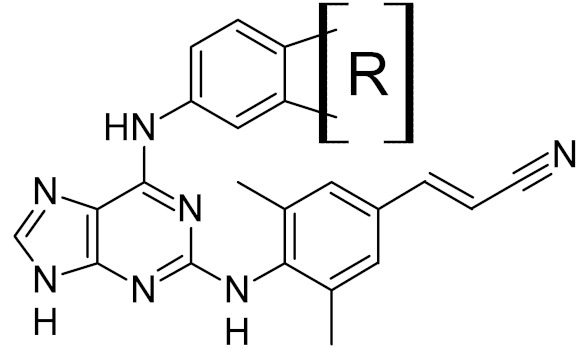	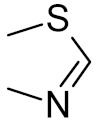			4.0	
	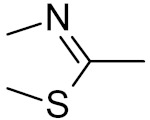			7.9	
	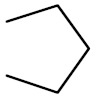			8.9	
	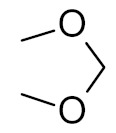			10	
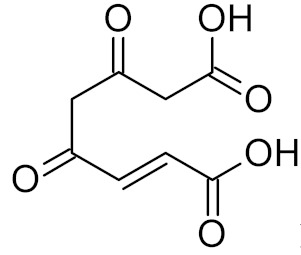 Fumarylacetoacetate		NEIL1	human	0.006	[77]
		NEIL2	human	0.032	
		OGG1	human	1.0	
		NTHL1	human	1.0	
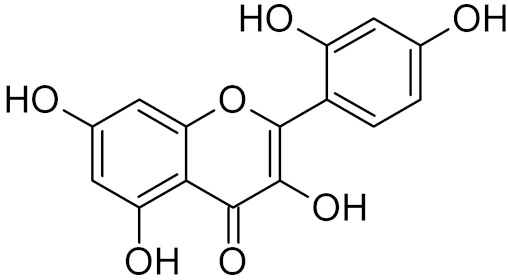 Morin		MPG	human	2.6	[78]

* IC_50_ for DNA polymerase activity in the presence of the D4/A20 complex; ** *K*_d_ or *K*_i_ directly measured; *** non-glycosylase member of the GO system (see Section 4 and Section 5).

**Table 3:** Known structures of DNA glycosylases bound to their inhibitors.

PDB ID	Enzyme	Inhibitor	Resolution, Å	Reference
6G3Y	OGG1	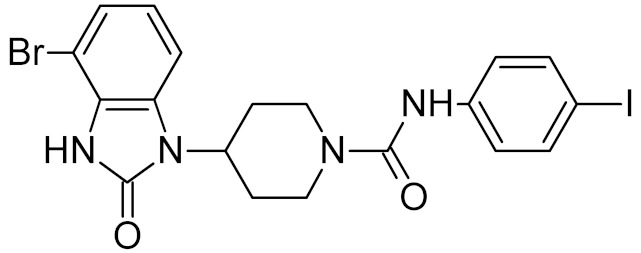	2.51	[73]
2HXM	UNG	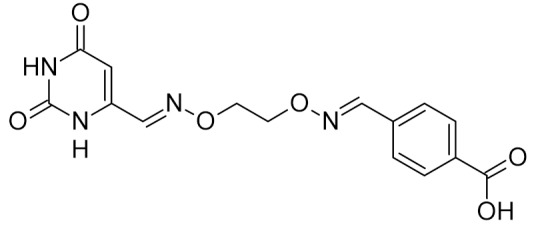	1.30	[62]
3FCF	UNG	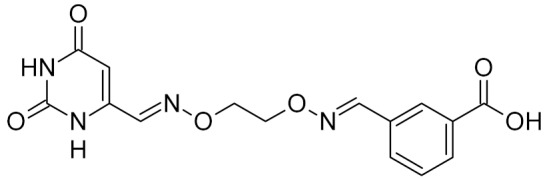	1.84	[61]
3FCI	UNG	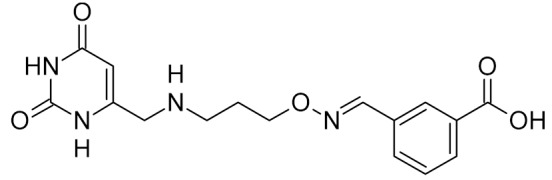	1.27	[61]
3FCK	UNG	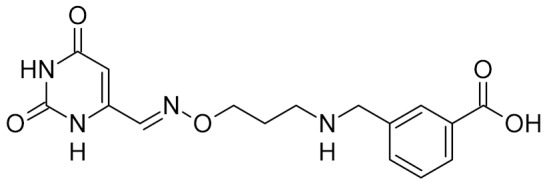	1.64	[61]
3FCL	UNG	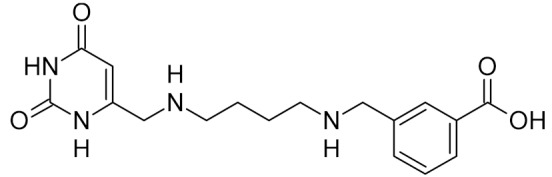	1.70	[61]
